# Value of Bark in Substance

**Published:** 1881-08-20

**Authors:** William A. Dayton


### VALUE OF BARK IN SUBSTANCE.

BY WILLIAM A. DAYTON, M. D.

It is well known that the sulphate of quinine, and especially i[illegible] [illegible] form of manufactured pills, varies in strength and efficacy—a fact due to the extent of its adulteration with cinchonidia and inert materials.

For this reason, no doubt, with me, many physicians have treated the acute infectious diseases often with embarrassing results—*i. e.*, patients would linger long beyond the time specified, when they expected to be well.

During the past six months I have used little or no sulphate of quinine. Remembering Professor Alonzo Clark's statement that "bark in substance often succeeds in breaking up a fever when quinia fails," I have accordingly used the bark prepared as follows, with results that fully warrant the exclusion of the sulphate of quinine from the list of drugs I prize most.

The bark *par excellence* to be used is that of the cinchona flava, one hundred grains of which yields from two to four grains of quinia alone, besides other alkaloids of equal, though disputed, efficacy.

In consequence of its occasional mixture with other barks, it is well for the physician to know the appearance of the genuine article when purchasing. For all intents and purposes, it is distinguished from the cinchona communis and rubra, and spurious barks: 1st, by its yellowish color; 2d, by its flattened, quill-shaped pieces; 3d, by its external (comparatively) smooth surface; 4th, by its fibrous fracture, with the escape of a fine powder; 5th, by its extremely bitter taste.

The bark selected with these precautions, and well bruised in an iron mortar, I use thus:

℞ Cort. cinch. flav.................................... ℥ iv.

Cort. aurant .......................................... ʒ ij.

Spts. vin. rect. dil.................................... Oj.

M. Allow to stand several hours, then percolate eight ounces.

The result is a fluid extract, of which a teaspoonful may be given three or four times daily.

As will be observed, several ounces of dilute alcohol remain in the percolator. This I express and use in future preparations, which, of course, are stronger than the first.

I have carefully compared the effects of this and Warburg's tincture; on the whole, the bark was most potent; and I believe that what is claimed for the latter is realized by the use of the former, viz: marked febrifuge effects in drachm doses, and tonic effects in smaller doses, at far less expense.

As in the case of quinine, so in the case of many other drugs, they are found to be so unreliable from manufacturers' doctoring, that the conscientious physician will soon be obliged to make his own preparations, and he will then get the eminently satisfactory results that therapeutists claim for materia medica.

It is well known that many physicians are losing favor because of occasional failures due to inferior drugs which are used in compounding prescriptions, besides the expense incurred; and I, for one, am inclined to accept the personal advice of one of our celebrities—none the less than Professor Willard Parker—and dispense and prepare my own drugs.—*N. Y. Medical Record.*

